# Impact of patients’ beliefs about insulin on acceptance and adherence to insulin therapy: a qualitative study in primary care

**DOI:** 10.1186/s12875-022-01627-9

**Published:** 2022-01-20

**Authors:** Changwei Liu, Jacqueline De Roza, Chai Wah Ooi, Blessy Koottappal Mathew, Elya, Wern Ee Tang

**Affiliations:** National Healthcare Group Polyclinics, 21 Geylang East Central, Singapore, 389707 Singapore

**Keywords:** Insulin adherence, Beliefs, Diabetes

## Abstract

**Background:**

Insulin therapy forms a cornerstone of pharmacological management of diabetes mellitus (DM). However, there remains a lack of acceptance and adherence to insulin, thereby contributing to poor DM control. This study aimed to determine the impact of patients’ beliefs about insulin on acceptance and adherence to insulin therapy.

**Method:**

This was a qualitative study using grounded theory approach. The study took place from September 2019 to January 2021 at a cluster of primary healthcare clinics in Singapore. Maximum variation sampling was used to recruit adult patients with type 2 DM on basal or premixed insulin for at least 6 months. Semistructured in-depth interviews were conducted using a topic guide and audio recorded. Data collection continued until saturation. Data analysis utilised a constant comparison procedure and a synthesis approach.

**Results:**

Twenty-one participants (mean age 61 years) were interviewed for this study. Data analyses showed that there were 6 main themes that emerged. Four themes influenced both insulin acceptance and adherence. These were concerns about insulin being a lifelong treatment, physical fear of insulin injection, erroneous beliefs about insulin, and perceived fear of DM complications. Two additional themes influenced adherence to insulin therapy. These were socioeconomic concerns, and concerns about side effects of insulin.

**Conclusions:**

Patients’ beliefs about insulin impact on the acceptance and adherence to insulin therapy. Health care providers need to elicit and address these beliefs during counselling to improve acceptance and adherence to insulin therapy.

## Introduction

The prevalence of diabetes mellitus (DM) is increasing worldwide and the complications of poorly controlled diabetes contribute to patients’ disabilities and rising health care costs [1–3]. In Singapore, the prevalence of diabetes in residents aged 18–69 rose from 7.3% in 1992 to 8.6% in 2017 [4]. By 2035, the prevalence of diabetes among Singapore residents is expected to be one in five [5].

The majority of patients with diabetes in Singapore have type 2 diabetes, which is influenced by modifiable lifestyle factors and characterised by insulin resistance along with diminished insulin secretion. The prevalence of type 2 diabetes has increased in tandem with the rising prevalence of obesity in Singapore, contributed to by excessive caloric intake and a sedentary lifestyle [6]. Nationwide screening initiatives and education campaigns to promote public awareness of diabetes also have led to greater detection of this condition [7].

Insulin therapy forms a cornerstone of the pharmacological management of diabetes. However, there remains a lack of acceptance of insulin initiation and adherence to insulin, thereby contributing to poor diabetes control [8]. In Singapore, the majority of patients with type 2 diabetes are diagnosed and managed in public and private primary care clinics. The public primary care sector consists of polyclinics, which manage 45% of patients with chronic diseases, including diabetes [9]. In polyclinics, a team of health care professionals, consisting of family physicians, advance practice nurses, nurse clinicians and allied health care staff, provides multi-disciplinary care to patients with diabetes. Despite the many opportunities for interaction with various health care professionals, insulin initiation and adherence remain a challenge.

It is hypothesised that beliefs on insulin influence the acceptance of insulin therapy [10, 11]. Concerns related to injection and negative beliefs such as insulin causing organ damage were barriers to insulin acceptance [12]. Fear of insulin side effects and embarrassment also negatively impacted adherence to insulin therapy [13]. In contrast, worries about complications of poorly controlled diabetes and beliefs that insulin was effective resulted in acceptance of insulin therapy [12]. Beliefs are influenced by the social-cultural context and dynamically shaped by experiences. There has not been a recent study done locally to explore this aspect; hence, this study aims to explore the impact of patients’ beliefs about insulin on acceptance of insulin initiation and adherence to insulin therapy.

## Methods

### Study design and setting

This was a qualitative study using a grounded theory approach. Semistructured individual interviews were conducted to elicit and explore patients’ beliefs on insulin and their impact on insulin initiation and adherence. The interviews provided content for data analysis.

Maximum variation sampling was used to recruit adult patients with type 2 diabetes mellitus on basal or premixed insulin for at least 6 months and followed up at National Healthcare Group Polyclinics. Sample size was determined by data saturation.

### Data collection

Potential participants were identified by health care professionals when they attended the clinics for regular follow-up visits and were referred to study team members for recruitment.

Semistructured in-depth interviews were conducted using a topic guide developed and based on a literature review and the clinical knowledge of the investigators. Patients were asked about their thoughts when they initially started on insulin and their experiences with insulin since. Their concerns about insulin, as well as their beliefs on the necessity of insulin, were explored in-depth.

The topic guide was revised if there were new points raised in the research. To avoid potential response bias, patients were not interviewed by health care providers who were involved in their care. There were 1 to 2 interviewers at each interview, and these interviews were carried out by health care professionals trained in conducting qualitative interviews. If a second interviewer was available, detailed notes and observations of nonverbal cues were taken down, and these were used as field notes.

From September 2019 to January 2021, 21 interviews were conducted; they were audio recorded and transcribed verbatim. Interviews were initially conducted face to face in clinics for patient convenience in private rooms that could be locked to ensure privacy and prevent intrusion. Recruitment was halted from February 2020 to June 2020 due to the COVID-19 pandemic and resumed in July 2020, with interviews conducted via a video conferencing Zoom platform.

### Analyses

Data analysis was performed by two coders and utilised a constant comparison procedure and a synthesis approach [14, 15]. A constant comparison procedure was used to develop and refine theoretically relevant concepts and categories [14].

The Lincoln and Guba framework for assessing the rigor of qualitative research included the factors of credibility, dependability, confirmability, transferability and authenticity [16]. In-depth interviews, audiotaping, verbatim transcription and data saturation enhanced the study’s credibility and authenticity. The development of a codebook and intercoder checks helped establish confirmability. Maximum variation sampling included a range of demographic and clinical characteristics that supported transferability.

This study was conducted in accordance with the Declaration of Helsinki. Written informed consent was obtained from all participants. Participant details were anonymised, and codes were used in their place. Hard copy and electronic data and audio recordings were stored in secured computers and locked cabinets accessible to study team members only. National Healthcare Group Domain Specific Review Board (DSRB) ethics approval was obtained for this study. (DSRB reference number: 2019/00096).

## Results

Twenty-one participants (mean age 61 years) were interviewed for this study (Table 1). Seventeen participants were interviewed by 1 interviewer, while 4 participants had 2 interviewers in their sessions. Data analyses showed that there were 6 main themes that emerged. Four themes influenced both insulin acceptance and adherence. These were concerns about insulin being a lifelong treatment, physical fear of insulin injection, erroneous beliefs about insulin, and perceived fear of DM complications. Two additional themes influenced adherence to insulin therapy. These were socioeconomic concerns, and concerns about side effects of insulin.

**Table 1:** Demographic and clinical information (*n* = 21)

**Age (years)** Mean (SD)	61.19 (10.06)
**Gender** (n, %)	
Male	11 (52.4)
Female	10 (47.6)
**Ethnicity** (n, %)	
Chinese	16 (76.2)
Malay	2 (9.5)
Indian	3 (14.3)
**Educational Level** (n, %)	
Primary education and below	10 (47.6)
Secondary education	9 (42.9)
Tertiary education	2 (9.5)
**Living Arrangement** (n, %)	
Alone	3 (14.3)
With family	18 (85.7)
**Employment Status** (n, %)	
Working	12 (57.1)
Not working	9 (42.9)
**DM Duration (years)** Mean (SD)	13.90 (8.36)
**Latest Hba1c (%)** Mean (SD)	8.27 (1.16)
**Insulin Duration (years)** Mean (SD)	5.94 (4.61)
**Insulin Regime** (n, %)	
Basal	9 (42.9)
Premix	12 (57.1)
**Insulin Device** (n, %)	
Vial and syringe	4 (19.0)
Pen	17 (81.0)
**Insulin Adherence**	
Fully adherent	12 (57.1)
Not fully adherent	9 (42.9)

### Theme 1: concerns about insulin being a lifelong treatment

There were concerns about insulin being a lifelong treatment, and this impacted on patient’s readiness to accept insulin initiation.

“*Because I heard that it's for life one that's why I don't want.”* (P01)

In fact, patients who thought insulin therapy was temporary were more accepting of it as part of their therapy.

*“Based on the advice from the doctor, I understand that ya. Moving forward, that will be the only way to go around these. I was also told that once my uh blood sugar stabilises and my HbA1c results become lower than uh 6, I may be off insulin. I was thinking that I should uh. stick uh, stick to the regime, uh, take my medication regularly, get some exercise and then uh, get off the insulin.”* (P14)

*“If my sugar level going down, maybe more than half a year get better right, of course the doctor will say "You can stop it."”* (P16)

### Theme 2: physical fear of insulin injection

Patients shared that the fear of needles and the pain on the thought of insulin injection also had been a barrier to accepting insulin when it was first started.

*“I very scared with the needle one. I dare not see also. You all prick me also I dare not see one. Nurse prick me also I dare not see.”* (P01)

*“I didn’t dare to jab it in, I really didn’t dare to jab it in at that time.”* (P03)

*“Because firstly I must say I really phobia on needle, you see.”* (P19)

*“It was like, the needle lo, before it even go in it was already painful.”* (P03)

*“Then, I was worried ah, worried if it will hurt during the injection.”* (P05)

*“Because I also very scared for injection on the body.”* (P20)

*“Actually, I have a bit phobia of using the insulin lah. Because you need to poke the needle into your stomach ah.”* (P21)

However, patients who were able to overcome this initial fear were able to incorporate insulin therapy as part of their daily routine.

*“Now I overcome already I now not scared already”* (P01)

*“It's a routine now”* (P01)

*“Then, I gradually, gradually start to adapt to it on my own lo. I have to overcome on my own, very relax, very relax, don’t think too much about it, very relax. Ah, then slowly I can do it already”* (P04)

### Theme 3: erroneous beliefs about insulin

There were multiple erroneous beliefs, largely from hearsay, on the purported side effects of insulin.

*“I also heard from my friends they were saying, take the jab, jab, jab already there will be many holes ah, you will have a lot of wind”* (P04)

*“I think the, after take the insulin ah, I definitely, 2 times ah, I think the sleep will have problem.”* (P13)

*“Sometimes, 1 week. I had, you know, constipation, like that. I observe that la, after insulin.”* (P19)

### Theme 4: perceived fear of DM complications

Patients’ perceived importance of DM control and the fear of DM complications motivated them to accept insulin therapy. Patients’ fear of experiencing the complications of diabetes spurred them to desire better diabetes control.

*“When it was high quite worried. I scared amputate the leg, Because my mum amputated.”* (P01)

*“If you don’t want the injection, as your condition (deteriorates) in the future, may lead to leg amputation, may become blind.”* (P11)

“Because you know why, because my wife also... also diabetes also. Also very high. In 5 days, he cut off the leg.” (P20)

*“Every day. I scared die already. That's why every day I don't miss my medicine.” (*P10)

*“Then later I realised I cannot skip coming here la, because when I measured it at home it was already 23, 24, I have the blood glucose metre at home la. Then I started thinking ah, cannot… Ah, then later I, I didn’t take the shots, the urinary there it’s very itchy. Then during sleep it was also like that, sticky and itchy la.”* (P05)

The belief that insulin therapy is necessary for controlling diabetes impacts patients’ willingness to accept insulin.

*“Because my condition requires injection. If don’t inject, it will keep going up and up.”* (P11)

*“At first, I was a bit hesitant and I said I don’t want injections. But the doctor told me that your diabetes level is very high. He said that only by taking insulin injections will I be able to manage and keep my diabetes under control.”* (P08)

When DM control improved with insulin therapy, it also reinforced patients’ motivation to continue with their therapy.

*“Now I find it so much better. I so happy now at least can go down to 1 digit.”* (P01)

*“After taking the shots it does go down.”* (P05)

*“Much more better (referring to DM control). I feel more relaxed.”* (P10*)*

*“He (doctor) said now your sugar is so good already. You got control.”* (P18)

*“After a few months, my HbA1c actually improved to 6.4. The good thing is, it will stabilise my blood sugar, the energy will be more or less ok.”* (P14)

*“Yah, so nowadays, these 2 years my blood sugar is much more controlled. Although it's not reached the, I mean, the normal level lah. Which is, but it's much more better than like, past number of years which shows it's quite bad.”* (P21)

### Theme 5: socioeconomic concerns

On the socioeconomic front, there were several concerns about insulin therapy. Cost concerns was one area that was highlighted.

*“Initially I was a bit scared that it will be too costly. At that time I also had another commitment, so there are a lot of things that have to be done ah. Ah, that equipment that test, test for the high blood ah. I said I don’t want to buy anything, all those extra commitments I don’t want. My own medical expenses already caused my finances to be very tight.”* (P04)

Cost concerns also led to patients’ inappropriate behaviour of reusing insulin needles as a coping mechanism which may potentially impact adherence.

*“Sometimes when I really want to save money hor, in the morning after using I won’t throw it away I still keep it for use at night leh.”* (P04)

*“Once is too wasteful. Throw after 1 use ah, I don’t have so much money, need to buy every time.”* (P11)

*“Because needle is I didn't change every day, is like a few days. Very costly. Because I'm not working and then a lot of things is like uh, quite expensive. Also, so I mean, use 1 time and you throw it, is like a bit waste, so might as well I use a few times. So I find that, I mean, I can use the needle a few days than I changing every day. Is like money throwing away every day. Like a dollar uh. The whole packet of 10, I think is $3 plus or $4 plus, I can't remember. But you divide into 30 days, of course it's cheaper right, but I find that I rather save it a bit, a few cents, I can accumulate a lot.”* (P16)

Patients who qualified for schemes that provided subsidies or reduced of out-of-pocket expenses were more accepting of the cost.

*“That one subsidised.” (referring to medications, injections, needles)* (P10)

*“Cost of insulin. Because now I’m paying by MediSave, so I don't feel anything yet*.” (P19)*“If the government did not issue that card, I won’t have any subsidy. It’s from welfare services.”* (P17)

In addition to economic costs, the busyness of daily life influenced adherence to the timing of insulin injection.

*“Sometimes ah, have, have to rush. After all the rushing it’s not very on time la, honestly speaking. We have to take care of the household, have to cook and have to buy vegetables also got to go to work.”* (P06)

Patients found that adhering to the evening dose of insulin could be challenging especially if they reached home late and forgot to administer the evening dose of insulin.

*“Sometimes I, you know, come, come back too late. Not often la. Sometimes I go out dinner. So maybe I forgotten.”* (P07)

*“When I’m out having some activities, sometimes usually.. sometimes I’ll be home late. Sometimes at night, I may miss it. Maybe when I reached home late after the activities, if I’m too tired.”* (P09)

*“Usually will forget the one at night la. Most of the time, when I eat out, I’ll forget la. feel that it’s very troublesome to bring along la, so don’t bring la. Forget already, don’t inject lor. Just take medicine only.”* (P15)

Shift work also was a factor that impacted compliance with the insulin regimen.

*“Shift duty. So that's why I didn't took because I didn't bring out to my workplace because at time will forget.”* (P03)

*“Sometimes I do night shift ah, I come back late ah, I don't take already. Cannot, cannot (inject at workplace) because uh, our workplace ah, is very dirty.”* (P20)

Patients found it inconvenient to inject insulin outside or in public toilets, and some patients were willing to administer their insulin only at home.

*“Every morning I had to do it at home already then I will go to work. Because over at our side it is considered a warehouse, the toilet is unisex. I don’t dare to take it at the toilets outside. Because it’s considered a public one, there’s also a long queue for it.”* (P04)

*“At night I do go out and eat also but I will eat outside and go back home to inject the insulin already. I don’t dare to take it at the toilets outside leh. It’s stressful ah, everyone is queuing for the toilet.”* (P04)

*“Sometimes when go out for dinner, some gathering with your friends, your family, don't bring the injection”* (P07)

However, patients did not have a similar perception of inconvenience in regard to taking oral medicines.

*“Very inconvenient when I’m out. more convenient for me to bring (oral) medicine along. You still need to find a place to administer the injection. You can’t just inject in public. So you take (oral) medicine hor, you can just take it out and eat.”* (P15)

### Theme 6: concerns about side effects of insulin

Patients expressed concerns about the side effects of insulin, especially that of hypoglycaemia episodes, which may potentially impact adherence if it occurs repeatedly.

“*Once the sugar level goes down, will start trembling badly. After you take the jab, you have to eat something immediately. Very quickly, the blood sugar level will go down.”* (P04)

*“Because sometimes when you take insulin, the effect can be a little faint-ish. When the sugar level becomes low, you will start to feel faint. When I first started taking the insulin medications, it used to happen. Once I feel faint, I will immediately eat something. Sometimes when I getting some work done and I forget to eat, I will start feeling faint. My arms and legs will start wobbling and I will feel weak. Once that happens, I will immediately eat something. Or I’ll quickly take a coca-cola from the refrigerator and drink.”* (P08)

*“Whole body is soaking wet and becomes blur blur. Already lost consciousness. You don’t know what you are doing.”* (P09)

*“Morning, bathe and eat early. I already know if do not eat, later I will be dizzy.”* (P12)

*“After injection, you forget to eat something, will just feel like, tsk, nervous la. And also dizzy la. And also, hands and feet feel cold. Trembling.”* (P15)

There also were concerns in relation to the process of insulin injection, such as pain or device issues that affect adherence.

*“There were a few times I didn’t take it. It was a torture at that time, when you try to poke in it hurts, once you poke, it hurts. Didn’t dare to poke it in too often.”* (P04)

*“Because sometimes injection smoothly, ok. Sometimes, once in a while ah, the thing doesn't move, you see, when you press the thing ah, the thing won't move ah, so I will hold it again and try to press. Don't know thing got stuck or what. Sometime cannot come out, I just pull the thing. Whole thing out. I don't inject already, because that time, I was so nervous and just gave up.”* (P19)

## Discussion

### Main findings and comparisons with existing literature

Patients have multiple concerns with regard to insulin, with their concerns stemming from the insulin itself as well as its administration. Insulin therapy is lifelong. The thought of insulin being a lifelong treatment is a barrier to insulin acceptance, and this also was demonstrated in a study by Arshad et al. among Pakistani patients, where the fear associated with lifelong commitment to insulin therapy was a significant barrier to insulin initiation [17]. Recognising insulin as a lifelong therapy also may be related to patients’ perceived chronicity of diabetes. Ledford et al. showed that while some patients understood that diabetes is a lifelong condition upon diagnosis, others did not accept its chronicity until after unsuccessful attempts at therapeutics or lifestyle modification, which could impact acceptance of treatment intensification [18].

With regard to the fear of needles and injection, studies overseas also have shown that this is not uncommon [19, 20]. Rubin et al., in a study of US adults, showed that the prevalence of fear of insulin injections ranged from 10 to 26%, and almost half shared that they would be more likely to take their insulin injections if a product were available to ease the pain [20].

On the socioeconomic front, Shafie et al. showed that cost is associated with patient adherence to insulin [21]. In our study, patients also were observed to be hesitant when asked why needles were reused, before sharing that it was due to financial concerns. This could reflect patients’ reluctance to discuss health care costs with care providers. Social factors such as the perceived stigma of injecting insulin in public areas and having to compromise on social activities in order to adhere to an insulin regimen, also have been demonstrated in other studies to impact on adherence to insulin therapy [22–26].

There were erroneous beliefs on insulin elicited in this study. Similarly, studies among African Americans also revealed erroneous perceptions, such as insulin causing organ damage [27, 28]. Ron Janes et al., in a qualitative study on patients in New Zealand, also showed that unscientific beliefs were also a barrier to good glycaemic control [11]. Worries about hypoglycaemia are a prominent concern for patients. Ellis et al. also showed that hypoglycaemia is a key barrier for patients, with an impact on their daily functioning and their engagement with insulin [19]. This does not only influences patients’ acceptance of insulin but also impacts health care providers’ decision to initiate insulin [29–31].

### Strengths and limitations

This qualitative study of patients’ beliefs on insulin therapy performed in the local primary care setting provides insight into the areas to focus on during insulin counselling. The emergence of COVID-19 infections in Singapore impacted on the ability to conduct face-to-face interviews, leading to a halt in patient recruitment starting in February 2020. However, the resumption of interviews from July 2020 using a video conferencing Zoom platform in accordance with safe distancing measures did not compromise the quality of interviews as video and audio quality was tested prior to each session. Although there were patients who declined participation due to their unfamiliarity with video conferencing, maximal variation sampling was eventually achieved. The study participants included patients with good and poor control of diabetes, enabling us to study their beliefs from various perspectives.

One potential limitation of this study is that perspectives from the health care providers were not explored. Patients’ beliefs about the acceptability of treatment may be different from those of health care providers [11]. This would be a relevant area to study in the local context, to see if there is a mismatch between the patient’s beliefs and what the health care provider perceives the patient’s beliefs to be. Correcting this mismatch would be important before engaging patients in a person-centred discussion of chronic disease management. A second limitation is that this qualitative study does not demonstrate the prevalence of these beliefs. Future quantitative research can examine the prevalence of these beliefs and their impact on insulin adherence.

### Implications for research and/or practice

The results show that patients’ beliefs about insulin impact acceptance and adherence to insulin therapy. The risks and benefits of insulin should be weighed in a patient-centred discussion to initiate insulin.

Erroneous beliefs about insulin should be corrected through tailored DM counselling once identified. Patients who feel that insulin therapy is temporary should be corrected for this erroneous belief, as this may lead to future nonadherence to insulin. Concerns about the side effects of insulin, such as hypoglycaemia, need to be elicited and addressed by health care providers during each review. Monitoring insulin use using video consulting services may enhance patient care with frequent checks for hypoglycaemia episodes.

A strategy to correct the fear of needles and injections would be to focus on showing how fine the needles are and reinforcing proper techniques to minimise pain. Health care providers should identify patients with true needle phobias and possibly apply graduated exposure therapy, handholding the patients through the process of injecting the needle to eventually overcome their phobias. Support groups, in which patients share how they overcome their fears, would be another useful avenue [32]. The costs of insulin therapy are about not only purchasing vials of insulin but also delivery devices and accessories for home blood glucose monitoring. Health care providers should proactively elicit financial concerns in patients and apply for subsidised schemes when necessary.

## Conclusion

This study explored the beliefs of patients with regard to insulin therapy. Issues such as the fear of side effects of insulin administration, cost concerns and patients’ perceived fear of DM complications are factors that influence insulin acceptance and adherence. Health care providers need to elicit and address these issues during counselling to improve acceptance and adherence to insulin therapy.

## Data Availability

The datasets used and/or analysed during the current study are available from the corresponding author on reasonable request.
